# Transcriptomic and Structural Insights into Leaf Variegation Development in *Ilex* × ‘Solar Flare’

**DOI:** 10.3390/ijms26093999

**Published:** 2025-04-23

**Authors:** Yiping Zou, Tao Zhuo, Yan Duan, Hong Chen, Peng Zhou, Mingzhuo Hao, Yunlong Yin, Donglin Zhang

**Affiliations:** 1 College of Forestry, Nanjing Forestry University, Nanjing 210037, China; yiping200889@126.com (Y.Z.);; 2 Institute of Botany, Jiangsu Province and Chinese Academy of Sciences (Nanjing Botanical Garden Memorial Sun Yat-Sen), Nanjing 210014, China; 3 Department of Horticulture, University of Georgia, Athens, GA 30602, USA; 4 College of Environment and Design, University of Georgia, Athens, GA 30602, USA; 5 Jiangsu Academy of Forestry, Nanjing 211153, China

**Keywords:** leaf variegation, pigment metabolism, chloroplast development, photosynthesis, transcriptome

## Abstract

The mechanisms underlying leaf variegation in the ornamental *Ilex* × ‘Solar Flare’ remain poorly understood. To investigate this phenomenon, we conducted a comprehensive characterization of its variegated leaves. Compared to green sectors, yellow sectors exhibited severe chloroplast structural abnormalities, including swollen chloroplasts, damaged thylakoid membranes, and reduced chloroplast numbers. These yellow sectors also showed significantly lower chlorophyll and carotenoid levels, along with a depletion of key chlorophyll precursors—protoporphyrin IX (Proto IX), magnesium protoporphyrin IX (Mg-Proto IX), and protochlorophyllide (Pchlide). Photosynthetic efficiency was significantly impaired. Comparative transcriptome analysis identified 3510 differentially expressed genes (DEGs) between yellow and green sectors. Key disruptions in chlorophyll biosynthesis included upregulated *CHLD* expression and downregulated *CHLH* and *CHLG* expression, leading to impaired chlorophyll synthesis. Additionally, chlorophyll degradation was accelerated by *PAO* upregulation. Defective chloroplast development in yellow sectors was associated with the downregulation of *GLK1*, *GLK2*, and thylakoid membrane-related genes (*PsbC*, *PsbO*, *PsbR*, *PsaD*, and *PsaH*). These molecular alterations likely drive the variegated phenotype of *I.* × ‘Solar Flare’. These observations advance our understanding of the genetic and physiological mechanisms regulating leaf variegation in this cultivar.

## 1. Introduction

Leaf variegation, characterized by distinct green and non-green sectors within the same leaf, represents a striking visual phenomenon that occurs across plant species [1]. This complex trait manifests primarily in herbaceous plants, with few documented cases in woody species [2]. Beyond its ornamental appeal—where variegation creates dynamic contrasts that enhance landscape esthetics [3]—this trait serves important ecological and physiological functions [4,5]. Recent studies suggest that variegation contributes to plant adaptation by influencing responses to abiotic factors, aiding in reproduction, and serving to protect against herbivores [1,6]. Additionally, variegation may help regulate leaf temperature through differential light absorption [7] and enhance ecological functions [8].

Variegated leaves are taxonomically classified into two principal categories: pigment-related variegation (including chlorophyll and other pigments) and structural variegation (resulting from epidermal modifications, air spaces, or appendages) [2]. Pigment-related variegation, the more common form, typically results from two primary mechanisms: impaired chloroplast development or mutations affecting chlorophyll metabolism [9]. Studies on model organisms, particularly *Arabidopsis* variegated mutants, such as *YELLOW VARIEGATED* (*var*)*1*, *var2*, and *var3*, have established incomplete chloroplast development as a fundamental driver of pigment-related variegation [10,11]. These mutants encode chloroplast homolog of *Escherichia coli* FtsH proteins, which are vital for maintaining chloroplast function and integrity [12,13]. Chloroplasts, the organelles responsible for photosynthesis, contain the thylakoid membrane system that houses the photosynthetic machinery [14]. Since chlorophyll biosynthesis occurs within chloroplasts, impaired chloroplast development often leads to a loss of leaf color. For example, mutations in *HvCMF7*, a gene essential for chloroplast biogenesis, impair chloroplast development and result in pigment-deficient or variegated leaf phenotypes in *albostrians* barley [15]. Similarly, the *immutans* (*im*) mutation in *A. thaliana* impairs chloroplast development, leading to reduced chlorophyll accumulation and compromised photosynthetic efficiency [16]. The *Golden 2-like* (*GLK*) transcription factor family, comprising *GLK1* and *GLK2* members, functions redundantly in photosynthetic tissues to regulate chloroplast biogenesis [17,18]. Overexpression studies have shown that the *Liriodendron* hybrid *LhGLK1* in *Arabidopsis* increases chlorophyll content, chloroplast number, and plant growth [19], while *BpGLK1* regulates chloroplast development and chlorophyll biosynthesis by interacting with the promoters of *BpCHLH* and *BpCRD1* genes [20].

Chlorophyll, the key photosynthetic pigment, plays a central role in determining leaf coloration and variegation patterns [21]. Its biosynthesis follows a complex pathway, beginning with the precursor 5-aminolevulinic acid (ALA) and progressing through numerous intermediates—including porphobilinogen (PBG), uroporphyrinogen III (Urogen III), coproporphyrinogen III (Coprogen III), protoporphyrin IX (Proto IX), magnesium-protoporphyrin IX (Mg-Proto IX), and protochlorophyllide (Pchlide)—before culminating in chlorophyll b and chlorophyll a production, via more than 20 enzymatic reactions [22]. Genetic alterations at any point in this pathway can disrupt chlorophyll biosynthesis, leading to different leaf color patterns [9]. For example, a frameshift mutation in the *OsCHLI* gene resulted in a chlorophyll-deficient yellow seedling phenotype with underdeveloped thylakoid membranes in rice [23]. Equally important to biosynthesis, chlorophyll degradation also significantly influences leaf coloration patterns. Key enzymes involved in chlorophyll catabolism include magnesium dechelatase (SGR), red chlorophyll catabolite reductase (RCCR), pheophorbidase (PPD), and pheophorbide *a* oxygenase (PAO) [24,25]. Studies have demonstrated that *CsSGR* overexpression in *A. thaliana* and *Nicotiana benthamiana* significantly reduced leaf chlorophyll content, indicating its role in regulating leaf coloration [26]. Therefore, chlorophyll loss in variegated leaves may stem from disrupted biosynthesis, accelerated degradation, or a combination of both processes.

Despite advances in high-throughput techniques such as transcriptome sequencing, proteomics, and metabolomics, the mechanisms underlying variegation remain poorly understood, particularly in woody plants. In model herbaceous species like *A. thaliana*, mutations such as *snowy cotyledon3* (*sco3-1*) mutants have been extensively studied using transcriptomic and genetic approaches, revealing that defective peroxisomal microtubule-associated protein involved in chloroplast development lead to leaf variegation [27]. However, in woody plants, such mechanistic understanding is still limited. For example, *Camellia sinensis* ‘Anji Baicha’, a tea cultivar with a temperature-sensitive albino phenotype, has been studied using transcriptomics and metabolomics to uncover DEGs and metabolites involved in chlorophyll biosynthesis, flavonoid metabolism, and stress responses [28,29]. Although informative, the complete regulatory network controlling the color change is still unclear. Similarly, in *Populus deltoides*, a yellow leaf mutant was analyzed using RNA-seq and physiological assays, revealing altered expression in genes associated with pigment metabolism and chloroplast function, yet the specific genetic mutations or regulatory hierarchies involved remain unidentified [30]. Members of the genus *Ilex* (holly), widely valued in urban landscaping, beverage production, traditional medicine, honey production, and timber industries, offer promising models for investigating variegation mechanisms in woody species [31,32]. Among these, *I.* × ‘Solar Flare’ exhibits stable, heritable variegation characterized by bright yellow margins surrounding green central regions. This distinctive and consistent phenotype makes it an ideal system for investigating the molecular and cellular basis of variegation in woody plants. Our study employs an integrative approach combining microscopic, biochemical, and transcriptomic approaches to uncover the mechanisms driving leaf variegation in *I.* × ‘Solar Flare’. Specifically, we aim to (1) characterize the structural differences between yellow and green leaf sectors at cellular and subcellular levels, (2) quantify pigment composition and photosynthetic capacity in each sector, and (3) identify key differentially expressed genes (DEGs) involved in chlorophyll biosynthesis, degradation, and chloroplast development.

Comparative analyses of yellow and green sectors revealed pronounced differences in cellular organization and function. Yellow sectors displayed abnormal chloroplast ultrastructure, significantly reduced pigment content, and compromised photosynthetic capacity. Transcriptome sequencing identified 3510 DEGs associated with pigment metabolism, chloroplast development, and photosynthetic function. Notably, we observed dysregulation of genes within chlorophyll biosynthesis (particularly *HEMB*, *CHLD*, *CHLH*, and *CHLG*) and degradation pathways (including *PAO*). Expression patterns of selected coloration-related genes were validated through quantitative real-time PCR (qRT-PCR). This comprehensive cytological, physiological, and molecular characterization provides a framework for understanding leaf variegation in *I.* × ‘Solar Flare’ and offers valuable insights that are applicable to other ornamental and woody species. Our findings advance both fundamental plant biology and applied horticulture, potentially informing strategies to enhance the environmental benefits and human welfare contributions of variegated plants.

## 2. Results

### 2.1. Varied Color Indices in VY

**Figure 1:**
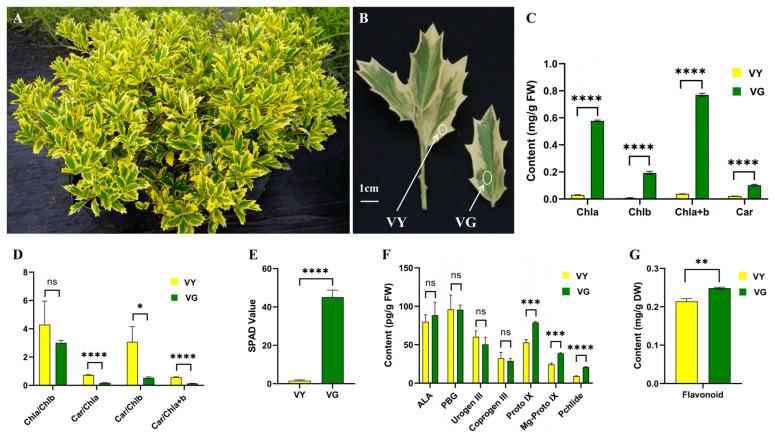
Leaf traits, pigment profiles, and chlorophyll precursors content in VY and VG. (**A**) *Ilex* × ‘Solar Flare’ plant. (**B**) Leaf traits of VY and VG. Scale bar = 1 cm. (**C**) Pigments content of VY and VG. (**D**) Pigments ratio of VY and VG. (**E**) SPAD value of VY and VG. (**F**) Chlorophyll precursors content of VY and VG. (**G**) Flavonoid content of VY and VG. The data are expressed as mean ± SD, with *t*-test statistical significance represented by: ns (not significant), *: *p* < 0.05, **: *p* < 0.01, ***: *p* < 0.001, ****: *p* < 0.0001. Abbreviations: ALA (5-aminolevulinic acid), Car (carotenoid), Chl (chlorophyll), Coprogen III (coproporphyrinogen), DW (dry weight), FW (fresh weight), Mg-Proto IX (Mg-protoporphyrin IX), PBG (porphobilinogen), Pchlide (protochlorophyllide), Proto IX (protoporphyrin IX), SD (standard deviation), SPAD (soil and plant analyzer development), Urogen III (uroporphyrinogen III), VG (the green sector of *Ilex* × ‘Solar Flare’), VY (the yellow sector of *I.* × ‘Solar Flare’).

The leaves of *I.* × ‘Solar Flare’ displayed a variegated phenotype, with green (VG) and yellow (VY) sectors (Figure 1A,B). The comparative analysis results of the color indices show that VG and VY have very significant differences in terms of L*, a*, b*, C, and H° (Table 1). The VY region showed significantly higher L* (75.17) than VG (46.76), indicating that VY is much lighter in color. The a* value was positive in VY (3.37) but negative in VG (−9.16), reflecting a shift from green in VG to a slightly reddish hue in VY. Similarly, the b* value, representing the yellow–blue axis, was substantially higher in VY (53.99) than in VG (27.02), confirming the strong yellow pigmentation of VY.

Additionally, C was markedly greater in VY (54.10) compared to VG (28.56), signifying higher color saturation in the yellow sections. In contrast, H° was significantly lower in VY (86.48) than in VG (104.40), indicating a shift toward a more intense yellow color in VY. These findings confirm that VG and VY are distinct in color properties, with VY exhibiting a brighter, more saturated yellow hue, while VG appears darker and greener.

### 2.2. Suppressed Pigment Synthesis in VY

Pigment analysis revealed that the chlorophyll a, chlorophyll b, chlorophyll a + b, soil and lant analyzer development (SPAD) value, carotenoid, and flavonoid were 19.51, 25.90, 20.79, 4.79, 28.81, and 1.16 times higher in green tissues (Figure 1C,E,G). Conversely, carotenoid/chlorophylls ratios were significantly elevated in yellow tissues, with carotenoid/chlorophyll a, carotenoid/chlorophyll b, and carotenoid/chlorophyll a + b, increasing by 4.07, 5.76, and 4.34 times, respectively. No statistically significant variation was observed in the chlorophyll a/chlorophyll b ratio (Figure 1D).

To understand the molecular basis of chlorophyll reduction, we analyzed key tetrapyrrole pathway intermediates (Figure 1F). ALA, PBG, Urogen III, and Coprogen III levels did not significantly differ between VY and VG, indicating functional early steps in the pathway. A major divergence was observed in the later steps of the chlorophyll biosynthetic pathway. Proto IX and Mg-Proto IX levels were significantly lower in VY, with a 33% reduction in Proto IX and lower Mg-Proto IX (24.42 vs. 38.77, *p* < 0.05). Pchlide levels were also lower in VY (9.16 vs. 20.97, *p* < 0.05) (Figure 1F). These results indicate a severe impairment in the later stages of chlorophyll biosynthesis, likely contributing to the reduced chlorophyll content observed in VY.

### 2.3. Reduced Stomatal Density and Disrupted Chloroplast Ultrastructure in VY

**Figure 2:**
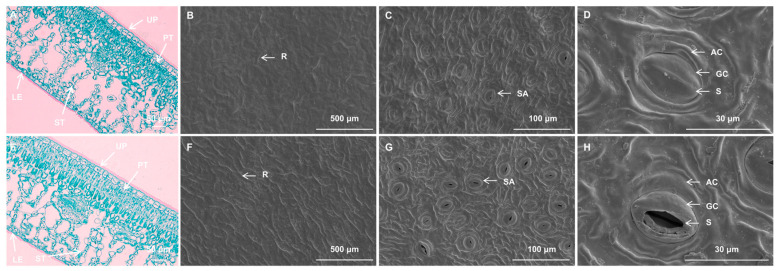
Comparative leaf anatomy and epidermal characteristics of VY and VG. Images (**A**–**D**) correspond to VY samples. Images (**E**–**H**) correspond to VG samples. (**A**) Anatomical structure of VY. (**B**) Upper epidermis of VY. (**C**,**D**) Lower epidermis of VY. (**E**) Anatomical structure of VG. (**F**) Upper epidermis of VG. (**G**,**H**) Lower epidermis of VG. Scale bars: (**B**,**F**) = 500 μm, (**C**,**G**) = 100 μm, (**A**,**E**) = 50 μm, (**D**,**H**) = 30 μm. Abbreviations: AC (arch cover), GC (guard cell), LE (lower epidermis), PT (palisade tissue), R (ridge), SA (stomatal apparatus), S (stoma), ST (spongy tissue), UP (upper epidermis), VG (the green sector of *Ilex* × ‘Solar Flare’), VY (the yellow sector of *I.* × ‘Solar Flare’).

Cross-sectional analysis showed uniform chlorophyll distribution throughout the mesophyll tissue in *I.* × ‘Conaf’ wild-type, while *I.* × ‘Solar Flare’ displayed uneven chlorophyll distribution with distinct yellow brim regions (Figure S1), matching its variegated phenotype (Figure 1A,B). Anatomical analysis of *I.* × ‘Solar Flare’ leaves showed similar structures in both yellow and green variegated regions. Both had a double-layered epidermis and distinct palisade and spongy tissues. The foliar structure displayed sun-adapted morphology, characterized by dual-layered palisade cells and nine layers of spongy cells arranged with considerable airspaces between them. While yellow and green regions had the same basic structure, the yellow regions were distinguished by their lower chloroplast concentration (Figure 2A,E).

Scanning electron microscopy analysis of *I.* × ‘Solar Flare’ yellow and green sections revealed similar epidermal micromorphological characteristics. Both leaf parts displayed inconspicuous epidermal cell margins with cuticle surface ridges, though yellow leaf tissues exhibited fewer ridges, and no epidermal hairs were observed (Figure 2B,F). Stomatal distribution was limited to the lower epidermis of the leaf, where they formed irregularly shaped oval complexes. Stomata are composed of two guard cells and two accessory cells, with outer arch covers having smooth inner margins (Figure 2C,D,G,H). While stomatal distribution and form were comparable between VY and VG, significant differences emerged: stomatal length and width remained consistent, but VY showed significantly lower stomatal density (Figure 2C,D,G,H and Table S1). Notably, VY exhibited a substantially higher percentage of closed stomata (84.47%) compared to VG (41.65%) (Table S1), suggesting impaired heat dissipation through stomatal mechanisms in VY.

**Figure 3:**
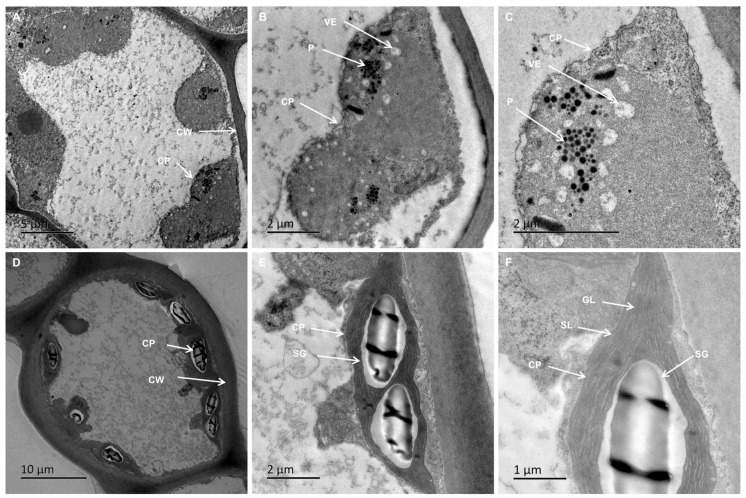
Chloroplast ultrastructure in VY and VG. Images (**A**–**C**) correspond to VY samples. Images (**D**–**F**) correspond to VG samples. Scale bars: (**D**) = 10 μm, (**A**) = 5 μm, (**B**,**C**,**E**) = 2 μm, (**F**) = 1 μm. Abbreviations: CP (chloroplast), CW (cell wall), GL (grana lamella), P (Plastoglobuli), SG (starch granule), SL (stroma lamella), VE (vesicle), VG (the green sector of *Ilex* × ‘Solar Flare’), VY (the yellow sector of *I.* × ‘Solar Flare’).

Chloroplast ultrastructure analysis revealed significant differences between VY and VG. VG chloroplasts exhibited spindle-shaped morphology with well-organized inner membranes, regularly distributed granal thylakoids, starch granules, and limited plastoglobuli, averaging 8.67 chloroplasts per cell (Figure 3D–F, Table 2). In contrast, VY chloroplasts appeared swollen, with absent or indistinct grana, few stromal lamellae, and dense populations of vesicles and plastoglobuli, reduced to approximately five chloroplasts per cell (Figure 3A–C, Table 2). Morphometric measurements showed VG chloroplasts at 6.78 × 4.01 μm (length/width ratio 1.69), while VY chloroplasts measured 4.77 × 4.29 μm (length/width ratio 1.12), indicating a more rounded shape in yellow regions (Figure 3A–C, Table 2). Notably, green sectors contained about 1.19 starch granules per chloroplast, whereas yellow sectors had none (Figure 3, Table 2), suggesting differences in carbohydrate storage capacity. These findings reveal significant modifications in chloroplast quantity, dimensions, and morphology, suggesting incomplete chloroplast development in yellow leaf sections.

### 2.4. Compromised Photosynthetic Capacity in VY

Photosynthetic analysis revealed significant impairment in yellow sections compared to green sections. Gas exchange parameters showed substantial reductions in VY, with net photosynthetic rate (P_n_), transpiration rate (E), and stomatal conductance (G_s_) decreased by 184.67%, 44.25%, and 40.14%, respectively, while intercellular CO_2_ concentration (C_i_) increased by 89.66% (Table 3). The negative P_n_ value in VY indicates a severe impairment in carbon fixation, where respiratory CO_2_ loss surpasses photosynthetic CO_2_ uptake, highlighting a fundamental dysfunction in the photosynthetic machinery. The decline in G_s_, coupled with the substantial increase in C_i_, suggests that although CO_2_ accumulates within the leaf tissues, it is not efficiently utilized for carbon assimilation. This further supports the notion that photosynthetic capacity in VY is significantly compromised, potentially due to disruptions in chloroplast function or metabolic limitations within the Calvin cycle.

Chlorophyll fluorescence parameters in VY also demonstrated marked decreases, with maximal quantum yield of PSII photochemistry (Fv/Fm), effective quantum yield of PSII (Y(II)), nonphotochemical quenching (NPQ), and regulatory energy dissipation quantum yield (Y(NPQ)) reduced by 77.14%, 74.47%, 90.48%, and 80%, respectively. In contrast, the nonregulatory energy dissipation quantum yield (Y(NO)) increased by 154.55% (Table 3). The drastic reduction in Fv/Fm suggests severe PSII photodamage, while the significantly lower Y(II) further confirms the reduced photochemical efficiency in VY. The sharp decline in NPQ indicates a weakened ability to dissipate excess light energy as heat, making VY more susceptible to photodamage. Additionally, the significant increase in Y(NO) reflects heightened photoinhibition and oxidative stress. The lower Y(NPQ) in VY further suggests compromised photoprotective mechanisms, exacerbating light-induced damage. These results collectively demonstrate severely compromised photosynthetic capacity in yellow sections.

### 2.5. Alterations in Gene Expression Related to Pigmentation and Light Harvesting Processes in VY

**Figure 4:**
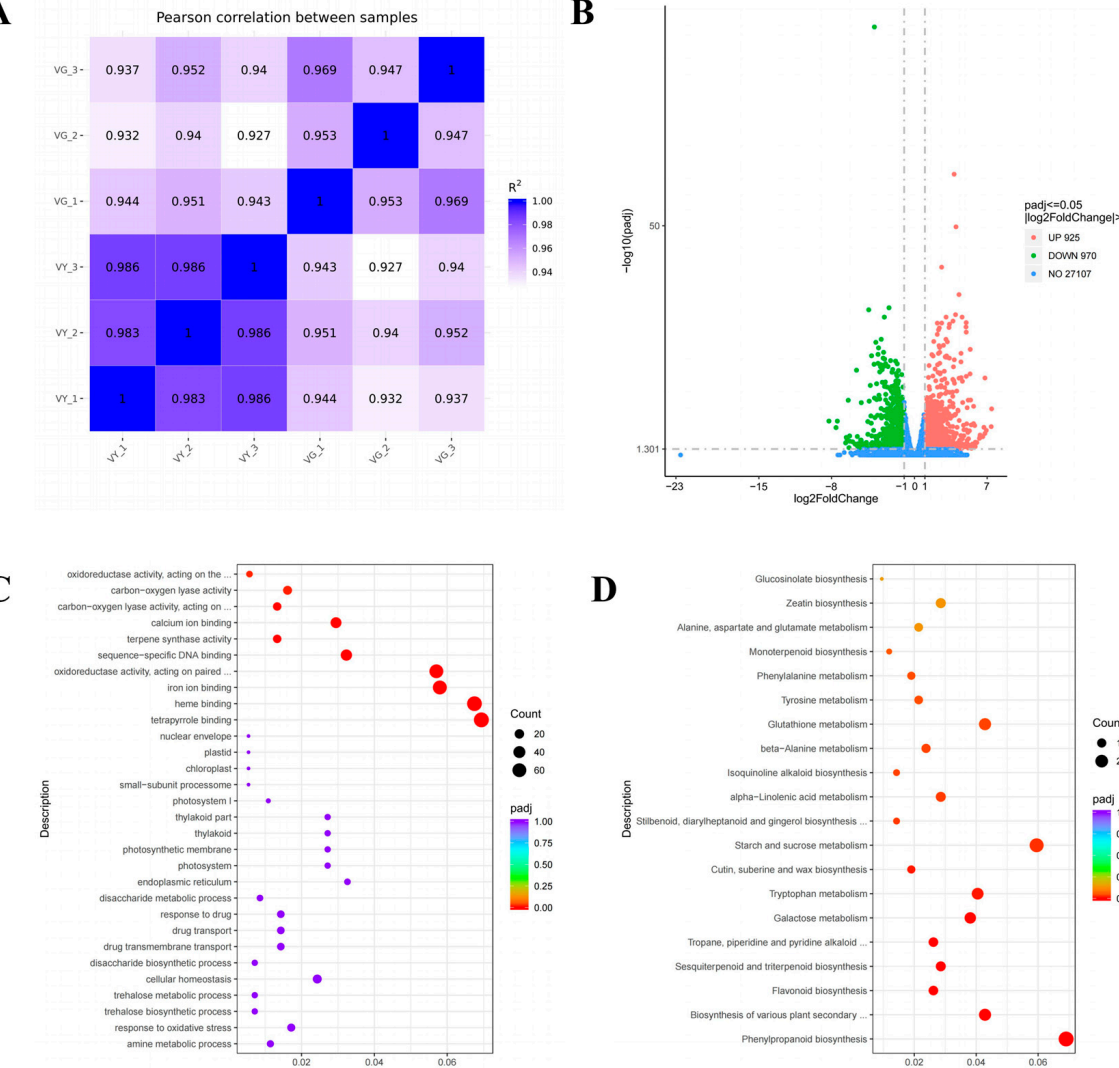
GO and KEGG enrichment analysis between VY and VG. (**A**) Pearson correlations. (**B**) Volcano plot distribution of DEGs. Log_2_ (fold change) plotted against −log_10_*Padj*, with upregulated transcripts highlighted in red and downregulated transcripts in green. (**C**) Top 30 enriched GO terms. (**D**) Top 20 enriched KEGG terms. The size of each bubble represents the number of associated DEGs, while color gradient (purple → blue → green → red) indicates increasing statistical significance (−log_10_*Padj*). Enrichment score is displayed on the horizontal axis. Note: VG (the green sector of *Ilex* × ‘Solar Flare’), VY (the yellow sector of *I.* × ‘Solar Flare’).

RNA-seq quality assessment produced 256,757,618 high-quality clean reads with Q20 and Q30 percentages above 96.72% and 91.26%, respectively, with GC content ranging from 43.66% to 44.54%. Alignment of the filtered data to the *I. latifolia* reference genome achieved mapping rates between 84.72% and 86.25% (Table S2). Data reliability was confirmed through high correlation values among biological replicates, as visualized by a heatmap of Pearson’s correlation coefficients and principal component analysis (Figure 4A). A comprehensive summary of the sequencing metrics is available in Table S3.

Comparative analysis between VY and VG identified 3510 DEGs, with 1573 showing increased expression and 1937 exhibiting reduced expression (Figure 4B). Functional classification through GO enrichment analysis highlighted 13 significantly enriched terms. Within the cellular component domain, thylakoid-related categories predominated, including “thylakoid” (GO:0009579), “thylakoid part” (GO:0044436), “photosynthetic membrane” (GO:0034357), and “photosystem” (GO:0009521). The molecular function category was primarily represented by terms related to pigment binding and metal ion interactions, such as “tetrapyrrole binding” (GO:0046906), “heme binding” (GO:0020037), and “iron ion binding” (GO:0005506) (Figure 4C and Table S4). The biological process category showed no statistically significant enrichment patterns.

Pathway analysis using KEGG annotation mapped the DEGs to 123 metabolic and signaling networks. Statistically significant enrichment (*Padj* < 0.05) was observed in pathways associated with secondary metabolism and photosynthesis, specifically “flavonoid biosynthesis” (ko00941), “nitrogen metabolism” (ko00910), and “photosynthesis–antenna proteins” (ko00196). Other notable enriched pathways included those involved in pigment production: “porphyrin metabolism” (ko00860) and “carotenoid biosynthesis” (ko00906) (Figure 4D and Table S4).

### 2.6. Altered Chlorophyll Biosynthesis Processes in VY

**Figure 5:**
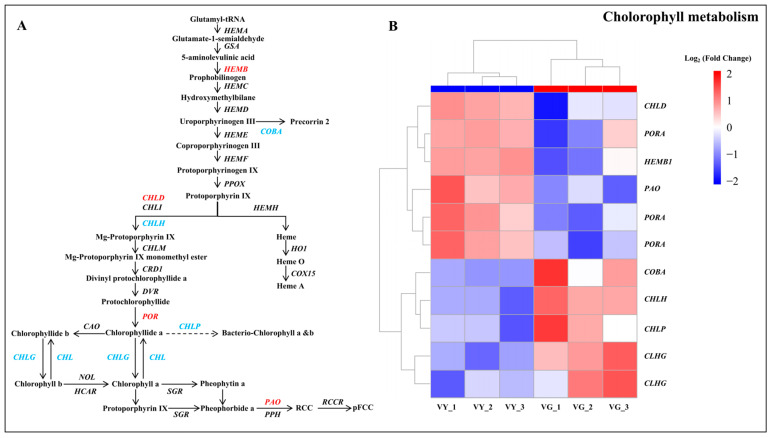
Chlorophyll metabolism pathway associated with DEGs between VY and VG. (**A**) Chlorophyll metabolism pathway. Upregulated genes are shown in red and downregulated genes in blue. (**B**) Heatmap of DEGs in chlorophyll metabolism pathway. Heatmap displays mean log_2_ (fold change) values calculated from three biological replicates per type with upregulated genes shown in red and downregulated genes in blue. Note: VG (the green sector of *Ilex* × ‘Solar Flare’), VY (the yellow sector of *I.* × ‘Solar Flare’).

Analysis of DEGs within chlorophyll metabolic pathways identified 11 genes with altered expression in the porphyrin and chlorophyll metabolism network (Figure 5 and Table S5). Among these, five genes related to chlorophyll synthesis (*CHLD*, *HEMB*, and three *PORA*) were upregulated, while five others (*CHLH*, *COBA*, *CHLP*, and two *CHLG*) were downregulated. Notably, the chlorophyll degradation gene *PAO* was also upregulated. Biochemical analysis revealed that early chlorophyll precursors maintained similar levels across both tissue types, whereas later intermediates (Proto IX, Mg-Proto IX, and Pchlide) were significantly depleted in yellow regions. This suggests an impediment in chlorophyll synthesis from Proto IX to Pchlide, potentially due to the downregulation of *CHLH*, *CHLG*, *CHLP*, and *COBA*, while the upregulation of *HEMB*, *CHLD*, *PORA*, and *PAO* further contributed to chlorophyll reduction.

GSEA highlighted markedly enriched molecular pathways in VY, including “flavone and flavonol biosynthesis” (ath00944), “porphyrin metabolism” (ath00860), and “oxidative phosphorylation” (ath00190) (nominal *p*-value < 0.05, NES > 1.5, FDR q-value < 0.25) (Table S6). The porphyrin metabolism pathway contained 28 additional DEGs, including *COX10*, *CHLI*, *SIRB*, *HO1*, *CHLD*, *PAO*, *HEMB*, *GSA*, *PORA*, *SGR*, and others with roles in tetrapyrrole metabolism (Figure S3 and Table S7). These comprehensive findings underscore the fundamental importance of coordinated chlorophyll biosynthesis and degradation in determining the distinctive variegation pattern of *I.* × ‘Solar Flare’.

### 2.7. Downregulated DEGs in Carotenoid and Flavonoid Biosynthesis in VY

**Figure 6:**
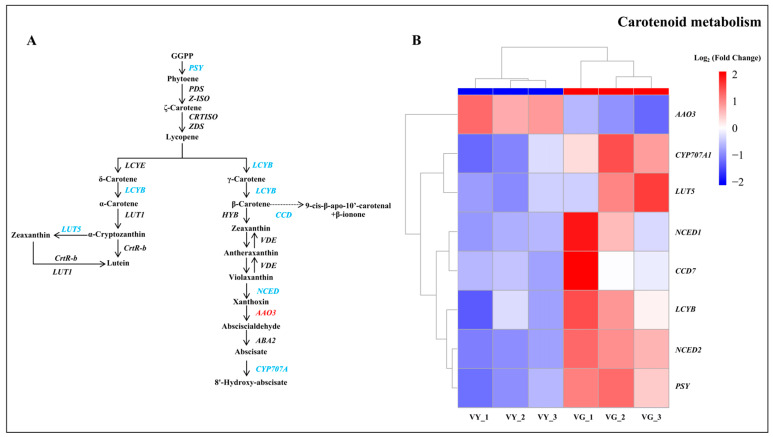
Carotenoid metabolism pathway DEGs between VY and VG. (**A**) Carotenoid metabolism pathway. Upregulated genes are shown in red and downregulated genes in blue. (**B**) Heatmap of DEGs in carotenoid metabolism pathway. Heatmap displays mean log_2_ (fold change) values calculated from three biological replicates per type with upregulated genes shown in red and downregulated genes in blue. Note: VG (the green sector of *Ilex* × ‘Solar Flare’), VY (the yellow sector of *I.* × ‘Solar Flare’).

Carotenoid biosynthesis analysis revealed eight DEGs, with seven downregulated in VY. Notable downregulated genes included *PSY*, *LUT5*, *NCED 1*, *NCED 2*, *LCYB*, and *CYP707A1*, while *AAO3* was significantly upregulated (Figure 6 and Table S5). The decreased carotenoid content in yellow regions correlates with the downregulation of carotenoid metabolism genes, suggesting disrupted biosynthesis pathways. Similarly, flavonoid biosynthesis pathway analysis identified 19 DEGs, with 12 downregulated and seven upregulated in the yellow leaf section, consistent with the observed reduction in flavonoid content (Figure S3 and Table S5).

### 2.8. Downregulated DEGs in Chloroplast Development and Photosynthesis in VY

Chloroplast development and division are determinant factors in leaf coloration. Analysis revealed downregulation of two DEGs (*GLK1* and *GLK2*) from the *Golden 2-like* (*GLK*) family, and one chloroplast-related gene (*LIR1*) in the yellow sections (VY), potentially explaining the impaired chloroplast development in these sections (Table S5).

**Figure 7:**
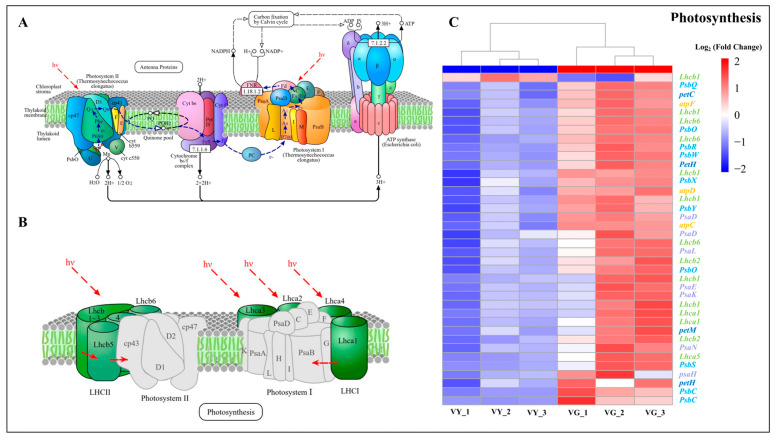
Transcriptional changes in photosynthetic machinery between VY and VG. (**A**) Schematic representation of the photosynthesis pathway. (**B**) Schematic representation of plant light-harvesting chlorophyll–protein complexes. (**C**) Expression heatmap of photosynthesis-related DEGs. Values represent mean log_2_ (fold change) calculated from triplicate samples of VY vs. VG. Upregulated genes appear in red; downregulated genes in blue. Note: VG (the green sector of *Ilex* × ‘Solar Flare’), VY (the yellow sector of *I.* × ‘Solar Flare’). Pathway diagrams in panels A and B were sourced from the KEGG database (https://www.kegg.jp, accessed on 10 January 2025).

Transcriptomic profiling of photosynthesis-related genes identified 41 DEGs between VY and VG sections (Table S5). The expression pattern revealed a striking imbalance, with only a single gene encoding photosynthesis–antenna proteins showing enhanced expression in VY. Conversely, 40 genes displayed significant downregulation across multiple photosynthetic components: PSII reaction center subunits, PSI reaction center subunits, cytochrome b6/f complex members, photosynthetic electron transport mediators, and F-type ATPase subunits (Figure 7 and Table S5). Among these, 18 downregulated genes are associated with the GO terms thylakoid, thylakoid part, and photosynthetic membrane, with five of them also classified under the thylakoid membrane GO term (Table S5). This pattern of downregulation may further explain the structural abnormalities observed in chloroplast development throughout these sections.

Among carbon fixation-related genes, 21 were downregulated and 3 upregulated. A similar regulatory pattern was observed in nitrogen metabolism, where 16 genes showed reduced transcript levels in yellow tissues, with just 1 gene exhibiting increased expression (Table S5). This widespread downregulation of photosynthesis-related genes suggests significant impairment of photosynthetic capacity in yellow sections.

### 2.9. Validation of Transcriptomic Data Through Quantitative Real-Time PCR (qRT-PCR)

To validate gene predictions from the transcriptomic analysis, we conducted quantitative real-time PCR (qRT-PCR) on 12 DEGs associated with chlorophyll metabolism, carotenoid biosynthesis, photosynthesis, and TFs, including *PORA*, *CHLD*, *CHLH*, *PAO*, *PSY*, *CYP707A1*, *PsaD*, *LHCA*, *PsbC*, *LHCB*, *petH*, and *GLK1* (Figure S4, Tables S1 and S4). In chlorophyll biosynthesis, *CHLD*, *PORA*, and *PAO* showed higher expression in VY than in VG, whereas *CHLH* was significantly downregulated. In carotenoid metabolism, *CYP707A1* and *PSY* were both downregulated in VY. Additionally, photosynthesis-related genes *LHCA*, *LHCB*, *petH*, *PsaD*, and *PsbC* exhibited lower expression in VY compared to VG. *GLK1*, a member of the *Golden 2-like* family, was also downregulated in VY. The high concordance between qRT-PCR expression patterns and RNA-seq profiles substantiated the reliability of our transcriptomic data, providing a solid foundation for subsequent functional analyses and biological conclusions.

## 3. Discussion

### 3.1. I. × ‘Solar Flare’ Categorizes Chlorophyll Deficiency Type Variegation

Our comprehensive analysis of *I.* × ‘Solar Flare’ leaf sectors reveals that its variegation mechanism belongs to the pigment-related category, specifically the chlorophyll deficiency type, according to Zhang et al.’s classification system [2]. While this classification appears straightforward, it has important implications for understanding the evolutionary and developmental mechanisms of variegation in woody ornamentals. Unlike structural variegation types that rely on physical properties for color differentiation (such as air-filled spaces in mesophyll tissue), the biochemical nature of *I.* × ‘Solar Flare’ variegation suggests a genetic or developmental regulation of pigment synthesis pathways [11,33], rather than from alterations in leaf anatomy development.

The similarity between *I.* × ‘Solar Flare’ and *I.* × *altaclerensis* ‘Belgica Aurea’ [34] suggests a potential common genetic basis for variegation within the *Ilex* genus. The conservation of cellular organization between green and yellow sectors in *I.* × ‘Solar Flare’ indicates that the variegation occurs at the chloroplast or molecular level rather than at the tissue development level—a characteristic that places this cultivar in line with well-studied variegation models like the *Arabidopsis var* and *im* mutants [13,33]. Understanding this categorization provides a crucial framework for interpreting our molecular findings and contextualizes *I.* × ‘Solar Flare’ within the broader spectrum of variegated plants.

### 3.2. Alterations in Pigment Metabolism DEGs May Contribute to the Leaf Variegation

Our transcriptomic and biochemical analyses revealed a coordinated disruption in pigment metabolism in the yellow sectors of *I.* × ‘Solar Flare’ leaves. This disruption is not merely a quantitative reduction in total pigment content, but reflects a selective impairment of the chlorophyll biosynthesis pathway. The disproportionate decrease in chlorophyll compared to carotenoids (20.79-fold vs. 4.79-fold) demonstrates a selective impairment of chlorophyll metabolism rather than a general suppression of all plastid pigment pathways. This selective reduction creates the distinctive yellow appearance through unmasking of carotenoid pigmentation, a phenomenon also observed in other variegated plants [35,36], but with notably greater severity in *I.* × ‘Solar Flare’.

The biosynthesis, cycling, and degradation of chlorophyll constitute a sophisticated metabolic network in plants, requiring the coordinated action of more than twenty different enzymes [37]. Analysis of chlorophyll precursors helps identify metabolic bottlenecks [38]. The metabolic bottleneck we identified in the Proto IX to Pchlide conversion represents a critical regulatory point in chlorophyll biosynthesis that differs from blockages reported in other variegated plants. While *B. peruviana* ‘Thimma’ shows impaired Proto IX synthesis [36], our findings demonstrate that *I.* × ‘Solar Flare’ variegation stems from disruptions downstream of Proto IX. This pattern suggests that the molecular lesion affects the Mg chelation step or subsequent conversions, rather than early tetrapyrrole synthesis. The strategic position of this blockage likely explains how *I.* × ‘Solar Flare’ maintains viable yellow sectors without risking phototoxic accumulation of early intermediates that can occur when earlier steps are blocked [37].

Differential expression of genes involved in chlorophyll biosynthesis further supports this interpretation. In the yellow sectors of *I.* × ‘Solar Flare’, five chlorophyll-related DEGs—*CHLH*, *COBA*, *CHLP*, and two *CHLG* homologs—were downregulated, while six—*CHLD*, *HEMB1*, *PAO*, and three *PORA*—were upregulated. Downregulation of genes like *POR*, *HEMA*, and *CAO* is known to cause yellowing and altered chloroplast structure in species such as *Hosta* [39]. Similarly, mutations in *DVR* cause yellow–green phenotypes in rice [40]. Among these, magnesium chelatase (MgCh), which inserts Mg^2+^ into Proto IX, is critical for chlorophyll biosynthesis [41,42]. This enzyme consists of three subunits (CHLD, CHLI, and CHLH), and the coordinated interaction of these subunits is essential for enzymatic activity [43]. Disruption of MgCh activity leads to decreased chlorophyll levels in leaf mutants [44]. For instance, *CHLI* suppression in strawberries led to yellow leaves [45], while a defective *GaCHLH* failed to properly interact with *GaCHLD* in *Gossypium arboreum* and impaired MgCh assembly, inhibiting chlorophyll synthesis [46]. In *I.* × ‘Solar Flare’, *CHLH* was downregulated while *CHLD* was upregulated in yellow tissues, likely impairing MgCh function and reducing chlorophyll biosynthesis.

Chlorophyll degradation also influences variegation [47]. The *PAO* is the primary mechanism for chlorophyll breakdown [48], beginning with chlorophyll b conversion to chlorophyll a, followed by chlorophyllase-mediated chlorophyll a degradation and Mg^2+^ removal by Mg-dechelatase [49,50]. Increased *PAO* expression correlates with reduced chlorophyll levels in the yellow leaves of *Ficus carica* and *Populus deltoides* [51,52]. As a key regulator of chlorophyll cycling [53], disruptions in *CHLG* expression can dramatically lower chlorophyll b content or prevent its formation entirely [47]. Earlier studies have demonstrated that diminished *CHLG* activity leads to lower chlorophyll accumulation [54]. In our study, *I.* × ‘Solar Flare’ exhibited suppressed chlorophyll biosynthetic genes and enhanced chlorophyll degradation in yellow tissues, leading to lower chlorophyll accumulation. Downregulation of *OsPORA* and *OsPORB* in rice causes yellow or white variegation [55]. Our study found increased *HEMB* and *POR* transcript levels, possibly as a compensatory response to disrupted chlorophyll biosynthesis, a phenomenon also seen in holly and tobacco leaf mutants [56,57]. Early-stage chlorophyll pathway mutations cause complete yellowing, whereas late-stage disruptions lead to variegation [58,59]. Our observed DEGs affected later synthesis stages, explaining the variegated phenotype in *I.* × ‘Solar Flare’.

Carotenoid function as auxiliary photosynthetic pigments and protectants against oxidative damage caused by light [60]. Research has demonstrated that mutations affecting carotenoid biosynthesis enzymes could produce variegated phenotypes [9]. The rice *zebra2* mutant, resulting from a defect in the *CRTISO* gene, displays pale green/yellow striping with reduced carotenoid levels concentration [61]. In *I.* × ‘Solar Flare’, seven DEGs involved in carotenoid synthesis were downregulated in yellow tissues, suggesting reduced carotenoid biosynthesis. Since carotenoid help stabilize chlorophyll levels [60], decreased carotenoid production may exacerbate chlorophyll reduction and the subsequent development of variegation.

### 3.3. Variegated Phenotype Was Linked to Abnormal Chloroplast Development

Chlorophyll synthesis occurs in three chloroplast regions: stroma (where ALA is converted to protochlorophyllide IX), chloroplast membrane (site of protochlorophyllide IX transformation to chlorophyllide), and thylakoid membrane (where final synthesis of chlorophyll a and chlorophyll b occurs) [62]. Genetic mutations that disrupt any of these chloroplast-localized processes often result in leaf variegation phenotypes [63]. For example, in tomatoes, a recessive mutation in the FtsH-like protein precursor caused chloroplast degradation and reduced chlorophyll levels in specific leaf sectors [64]. In sunflowers, point mutations in the *psaA* gene disrupted photosynthetic electron transport, leading to chlorophyll deficiency and poorly developed thylakoid membranes, resulting in yellow leaf sectors [65]. Similarly, silencing of *PDC*-*E1β* genes in tobacco led to a pale green variegated phenotype due to impaired chloroplast development [66]. Chloroplasts, which are responsible for photosynthesis [67], contain essential photosynthetic complexes embedded in the thylakoid membrane [68]. Disruptions in thylakoid organization can severely affect chloroplast development and photosynthetic efficiency [69]. Our transcriptomic analysis identified 40 downregulated DEGs associated with photosynthetic processes, of which, 18 were related to thylakoid function. This suggests a pronounced suppression of thylakoid membrane development and reduced photosynthetic efficiency, consistent with findings in other variegated plants [36,61].

*GLK* transcription factors play a crucial role in chloroplast biogenesis and chlorophyll metabolism by regulating genes involved in these processes [70]. They initiate plastid differentiation in response to light [71], and overexpression of *GLK* genes enhances chloroplast formation [72]. In maize, *ZmGLK1* regulates chloroplast differentiation in bundle sheath cells [73], while overexpression of *SlGLK2* in tomatoes increases plastid count and pigment level [74]. In lettuce, a CACTA transposon insertion in *LsGLK* altered splicing patterns, leading to a transition from dark to pale green leaves [75]. In our study, both *GLK1* and *GLK2* were downregulated, consistent with findings in studies on two other holly leaf mutants [57,76], as well as variegated *B. peruviana* [36] and *Osmanthus fragrans* [77]. TEM revealed stark differences between chloroplast ultrastructure in green and yellow leaf sectors. Green-sector chloroplasts had well-organized thylakoid systems, indicative of functional photosynthesis. In contrast, yellow-sector chloroplasts exhibited severe structural defects, including swollen shapes, indistinct or absent grana, fewer stromal lamellae, and increased vesicles and plastoglobuli. These abnormalities correlated with lower chlorophyll content, photosynthetic efficiency, stomatal conductance, and maximum photosynthetic rate, similar to findings in variegated crabapple [78] and *Ginkgo biloba* [79].

The relationship between impaired chloroplast development and reduced chlorophyll content appears to be bidirectional. Chlorophyll synthesis occurs within chloroplasts and requires functional chloroplast compartmentalization [62]. Conversely, chlorophyll is essential for the stabilization of photosynthetic complexes and thylakoid membrane organization [36]. This interdependence creates a potentially self-reinforcing cycle wherein defects in chloroplast development compromise chlorophyll synthesis and reduced chlorophyll levels further impair chloroplast development, ultimately leading to the stable yellow variegation observed in *I.* × ‘Solar Flare’.

## 4. Materials and Methods

### 4.1. Plant Materials

The holly plant *I.* × ‘Solar Flare’ exhibits distinctive foliage with a green center (VG) and yellow brim foliage (VY) (Figure 1A,B). The study utilized 100 four-year-old cutting-grown liners cultivated in 3-gallon containers with a peat–perlite substrate (3:1 by volume, pH 6.5) at Nanjing Qingzhuguo Landscape Horticulture Co., Ltd., Nanjing, China (32°13′ N, 118°49′ E). Leaf samples were collected in June 2023, and the green and yellow tissues were separated with scissors, immediately immersed in liquid nitrogen, and maintained at −80 °C for future examination.

### 4.2. Measurement of Leaf Color Indice and SPAD

Leaf color analysis was performed using a CR-8 precision color measuring instrument (Shenzhen 3nh Technology Co., Ltd., Shenzhen, China), with 10 leaves measured in triplicate. The color parameters measured were L* (lightness, 0 = black, 100 = white), a* (red-green axis, +a* = red, −a* = green), and b* (yellow-blue axis, +b* = yellow, −b* = blue) [80]. Additional indices were calculated as follows: H° = arctan (b*/a*), a*/b*, and C = (a*^2^ + b*^2^)^1/2^. The parameter H° describes color (red = 0°, yellow = 90°, green = 180°, blue = 360°), while C* represents color intensity [81]. A SPAD-502 Chl meter (Konica Minolta Sensing, Tokyo, Japan) was used to measure the SPAD level. Values were recorded from three randomized positions across the apex, midsection, and base of each leaf, with final data representing averages from three biological replicates.

### 4.3. Analysis of Chlorophyll, Carotenoid, Flavonoid, and Chlorophyll Precursors Levels

Chlorophyll a, chlorophyll b, and carotenoid content were quantified using the method described by Gao et al. [29]. Fresh leaves (20 mg) were excised, finely cut into filaments, and thoroughly homogenized. The tissue was then extracted in 5 mL of 95% ethanol and incubated at room temperature for 24 h under dark conditions. Following extraction, the supernatant was carefully collected, and absorbance measurements were performed at 665 nm, 649 nm, and 470 nm using a UV-2550 spectrophotometer (Shimadzu, Kyoto, Japan). Pigment concentrations were calculated using the following equations:

Chlorophyll a (Ca) = 13.95 × A_665_ − 6.88 × A_649_

Chlorophyll b (Cb) = 24.96 × A_649_ − 7.32 × A_665_

Total chlorophyll (Ct) = Ca + Cb

Carotenoids (Cx + c) = (1000 × A_470_ − 2.05 × Ca − 114.8 × Cb)/245

where A_665_, A_649_, and A_470_ represent the absorbance values at 665 nm, 649 nm, and 470 nm, respectively. The pigment content per unit of fresh weight was calculated as:

Pigment content (mg·g^−1^ FW) = [pigment concentration (mg·L^−1^) × extract volume (L) × dilution factor]/sample fresh weight (g)

Flavonoid content was measured according to a previously established method [82]. A total of 0.5 mL of diluted extract or standard solution was mixed with 2 mL double-distilled water in 15 mL conical tubes. Then, 0.15 mL of 5% NaNO_2_ solution was added and reacted for 5 min. Then, 0.15 mL of 10% AlCl_3_·6H_2_O solution was added, and it was incubated for 5 min. Finally, 1 mL of 1 M NaOH solution was added, the mixture was vortexed thoroughly, and incubated for 15 min. Absorbance was measured at 415 nm. Total flavonoid content was determined based on a rutin standard curve:

A = 0.01069C − 0.001163, r = 0.9998

where A is the absorbance and C is the flavonoid content in mg·g^−1^.

Chlorophyll precursor analysis was conducted by following the method described by Zou et al. [57]. Fresh leaf samples (50 mg) were ground in liquid nitrogen, and extracted in 450 mL of 0.01 mol L^−1^ PBS solution (pH 7.4). Samples were centrifuged at 12,000 rpm for 10 min to obtain the supernatant, which was subsequently subjected to plant ELISA (#YJ503247, Yuanju, Shanghai, China) to determine levels of seven chlorophyll biosynthetic intermediates: ALA, PBG, Urogen III, Coprogen III, Proto IX, Mg-Proto IX, and Pchlide. Three biological replicates were performed for both leaf samples.

### 4.4. Observation of Leaf Anatomical Structure

Fresh green leaves from wild-type *I.* × ‘Conaf’ and variegated *I.* × ‘Solar Flare’ were cross-sectioned. Green and yellow leaf tissues from *I.* × ‘Solar Flare’ were cut into rectangular sections (2 cm × 1 cm) and fixed using a preservative mixture containing formalin, alcohol, and glacial acetic acid mixed at 90:5:5 volumetric proportions. The samples remained in this fixative for at least 48 h. Following the fixation step, the tissue underwent a progressive dehydration process through an ethanol gradient series (70%, 80%, 90%, 95%, 100%, 100%; 2 h each). The dehydrated samples were then cleared with two sequential xylene baths (2 × 10 min) before being embedded in paraffin wax, following standard procedures described in reference [83]. After embedding, samples were sectioned at 8–10 μm thickness using a rotary microtome and mounted on glass slides coated with Mayer’s albumin adhesive. The sections underwent a dual staining procedure using 1% aqueous safranin (30–60 min) followed by 0.5% fast green in 95% ethanol (30 s) as a contrast stain. Following dehydration through an ascending ethanol series and clearing in xylene, slides were mounted with DPX medium. Images of the prepared sections were obtained with an Olympus BX53 light microscope (Olympus Corporation, Tokyo, Japan) equipped with a digital camera, and anatomical parameters including leaf thickness.

### 4.5. Observation of Leaf Epidermis Microstructure

Leaf epidermal microstructure was analyzed using cryogenic scanning electron microscopy (CryoSEM), as detailed previously [84]. Fresh, mature leaves were cut into 1 cm × 1 cm fragments, avoiding major veins, and mounted on 4 cm × 2 cm copper plates using thermal compound paste. Care was taken to ensure proper orientation with adaxial or abaxial surfaces facing upward for respective analyses. After freezing at −20 °C for 30 min to preserve cellular structure, the samples were transferred to a sputter coater where they were gold-coated (approximately 15 nm thickness) in an argon environment at 30 mA for 60 s. Analysis was performed with a Quanta 200 scanning electron microscope (FEI, Hillsboro, OR, USA), equipped with a Deben Coolstage refrigerating unit (Deben, Suffolk, UK) at −30 °C throughout the observation period. The scanning electron microscopy was conducted under high vacuum conditions with an accelerating voltage of 20 kV, utilizing a backscattered electron detector positioned at a working distance of 8–12 mm from the specimen surface. For each sample, at least five different fields were examined and photographed at various magnifications (200×, 600×, and 3000×) to document stomata, and other epidermal features, with measurements of stomatal dimensions taken from minimum 10 stomata per sample.

### 4.6. Observation of Chloroplast Ultrastructure

Using standard transmission electron microscopy (TEM) protocol, leaf tissue was sliced into 2 mm × 1 mm fragments and subsequently fixed for 2 h at room temperature in 2.5% glutaraldehyde solution prepared with 0.1 mol L^−1^ phosphate buffer (pH 7.2) [85]. After triple washing with phosphate buffer, samples underwent post-fixation in 1% OsO4 for 7 h, followed by three additional buffer washes. The specimens underwent dehydration through an ascending acetone gradient (30%, 50%, 70%, 80%, 95%, and 100%, 15–20 min per concentration), followed by elution with acetone–ethanol mixtures (3:1, 1:1, 1:3) for 0.5 h each, and a final acetone wash for 1 h. Infiltration was performed using a series of acetone resin gradients with sequential incubations of 2–4 h, overnight, and 2–4 h. The process concluded with embedding in pure resin and polymerization at 65 °C for 48 h. Sections of 50–80 nm thickness were prepared using a Leica UC6/FC6 ultra-microtome, stained with lead citrate and 2% uranium acetate for 8 min, and analyzed with a JEM 1400 transmission electron microscope (JEOL Ltd., Tokyo, Japan). To quantify chloroplasts, we counted their numbers in 10 random cells and measured the dimensions of 10 intact chloroplasts, ensuring that each measured chloroplast came from a different cell.

### 4.7. Analysis of Photosynthetic and Chlorophyll Fluorescence

Photosynthetic parameters (net photosynthetic rate—Pn, stomatal conductance—Gs, intercellular CO_2_ concentration—C_i_, and transpiration rate—E) were measured using a LI-6400 portable system (LICOR, Lincoln, NE, USA) between 09:00 and 11:00 on clear days. The chamber temperature was maintained at 25 °C, CO_2_ concentration at 400 μmol mol^−1^, and light intensity at 1000 μmol m^−2^ s^−1^.

Chlorophyll fluorescence parameters were assessed with a pulse amplitude modulation (PAM) fluorometer (Junior-PAM-II, Heinz Walz GmbH, Effeltrich, Germany) between 10:00 and 12:00 on the same day, following a published method [57]. Initial fluorescence (Fo) and maximum fluorescence (Fm) were measured after 30 min of dark acclimation followed by 15 min of rest, indicating fluorescence yields at fully open and closed PSII reaction centers, respectively. Parameters such as maximum quantum yield (Fv/Fm), actual photosynthetic efficiency (Y(II)), non-photochemical quenching (NPQ), regulatory energy dissipation quantum yield (Y(NPQ)), and non-regulatory energy dissipation quantum yield (Y(NO)) were determined using WinControl-3 software (version 3.34). For each biological replicate of yellow and green leaf regions, measurements were taken from three leaves of three randomly selected plants.

### 4.8. RNA Extraction and cDNA Library Preparation

The de novo transcriptome of VY and VG of *I.* × ‘Solar Flare’ plants was assembled. Total RNA was extracted from yellow and green leaf samples using the NEBNext^®^ Ultra^TM^ Plant RNA Extraction Kit (TaKaRa, Dalian, China), following the previously described method [57]. The quality and integrity of extracted RNA were assessed through multiple methods: 1% agarose gel electrophoresis, a Nanodrop 1000 spectrophotometer (Nanodrop, Wilmington, DE, USA) to evaluate A260/A280 ratios, and an Agilent 2100 Bioanalyzer (Agilent Technologies, Inc., Santa Clara, CA, USA) for concentration and RNA integrity number (RIN) determination, with samples exhibiting RIN values ≥ 8.0 selected for further processing. For cDNA library construction, mRNA was isolated from qualified total RNA samples using the NEBNext Poly(A) mRNA Magnetic Isolation Module (E7490; NEB, Ipswich, MA, USA), then fragmented into 200 nt inserts for cDNA synthesis. First-strand cDNA was synthesized using random hexamer primers and reverse transcriptase, while second-strand cDNA was generated using DNA polymerase I and RNase H. The resulting double-stranded cDNA underwent end-repair, dA-tailing, adaptor ligation, and PCR amplification to enrich the fragments. The quality of the resulting libraries was evaluated using the Agilent 2100 Bioanalyzer, and the concentration was determined by qPCR. Equal amounts of the indexed libraries were pooled and sequenced on the Illumina NovaSeq 6000 platform (Illumina, San Diego, CA, USA) to generate 150 bp paired-end reads for downstream transcriptome assembly.

### 4.9. Illumina Deep Sequencing and Bioinformatics Analysis

Library preparation was performed according to Illumina TruSeq protocols, with DNA fragmentation to 350 bp average insert size. Sequencing of cDNA libraries was performed on the Illumina NovaSeq 6000 platform (Illumina, San Diego, CA, USA), generating approximately 30 million paired-end reads (2 × 150 bp) per sample. Raw sequencing data underwent quality control using custom Perl scripts to eliminate N bases, adaptors, and low-quality reads, followed by additional quality filtering with Trimmomatic (v0.39). The quality of the clean data was evaluated based on Q20, Q30, and GC content using FastQC (v0.11.9). Clean reads were aligned to the *I. latifolia* reference genome (https://ngdc.cncb.ac.cn/gwh, accessed on 10 November 2024, GWHBIST00000000) using HISAT2 (version 2.0.4). HTSeq (version 0.6.1) was employed to quantify gene expression, with results reported as FPKM (fragments per kilobase of transcript per million mapped reads).

### 4.10. Identification and Functional Analysis of DEGs

Differential gene expression analysis was carried out using DESeq2 R software (version 1.20.0), employing a negative binomial distribution model. Transcripts with adjusted *p*-values (*Padj*) ≤ 0.05 and |log_2_(Fold Change)| ≥ 1 were classified as DEGs. Functional annotation was conducted through Gene Ontology (GO) and Kyoto Encyclopedia of Genes and Genomes (KEGG) enrichment analyses via the cluster Profiler R package (version 4.16.0). Significantly enriched GO terms were determined using a hypergeometric test with Benjamini–Hochberg correction (adjusted *p* < 0.05) to categorize DEGs into biological process, molecular function, and cellular component terms. KEGG pathway analysis was conducted to identify significantly enriched pathways (adjusted *p* < 0.05) using the same statistical framework. Gene set enrichment analysis (GSEA) was conducted using the local analysis tool (http://www.broadinstitute.org/gsea/index.jsp, accessed on 20 November 2024) with the KEGG dataset to identify significant and consistent differences in predefined gene sets, revealing characteristic enrichment patterns across both downregulated and upregulated DEGs in all pairwise comparisons.

### 4.11. qRT-PCR Verification

RNA-seq results were validated through quantitative real-time PCR (qRT-PCR) of 12 randomly selected genes, using *Actin* as the internal control reference (Chong et al. [86]). Total RNA was extracted from samples using the RNAprep Pure Plant Plus Kit (#DP432, Tiangen, Beijing, China), with RNA quality assessed via spectrophotometry (A260/A280 ratio ~2.0) and gel electrophoresis. First-strand cDNA synthesis was performed using the HiScript III 1st Strand cDNA Synthesis Kit (#R312-01, Vazyme, Nanjing, China), according to manufacturer’s instructions. Gene-specific primers were designed using Primer Premier 6.0 software (Premier Biosoft Inc., San Francisco, CA, USA) with amplicon sizes ranging from 70 to 150 bp (Table S8). The qPCR reactions were conducted using AceQ Universal SYBR qPCR Master Mix (#Q511-02, Vazyme, Nanjing, China), with each reaction containing 10 μL SYBR Green Master Mix, 0.5 μM of each primer, and 5 μL of diluted cDNA template. Amplification was performed on a StepOnePlus real-time PCR system with initial denaturation at 95 °C for 10 min, followed by 40 cycles of 95 °C for 15 s and 60 °C for 60 s, with melting curve analysis to confirm specific amplification. Each analysis was conducted using three biological replicates, with each sample further divided into three technical replicates to ensure reliability. Expression levels were normalized to *Actin* [86] using the 2^−ΔΔCt^ method to determine comparative expression levels [87].

### 4.12. Statistical Analysis

Data analysis was performed using SPSS 19.0 (SPSS Inc., Chicago, IL, USA) for statistical evaluations and GraphPad Prism 9.0 (GraphPad Inc., La Jolla, CA, USA) for figure generation. Significance testing relied on one-way ANOVA and *t*-test methods, with results achieving *p*-values below 0.05 deemed statistically significant. All quantitative data appear as mean ± SD throughout the manuscript. Visual data were analyzed using ImageJ software (https://imagej.net/ij/, accessed on 25 November 2024), and cytological measurements were obtained from 10 microscopic fields selected at random for evaluation.

## 5. Conclusions

**Figure 8:**
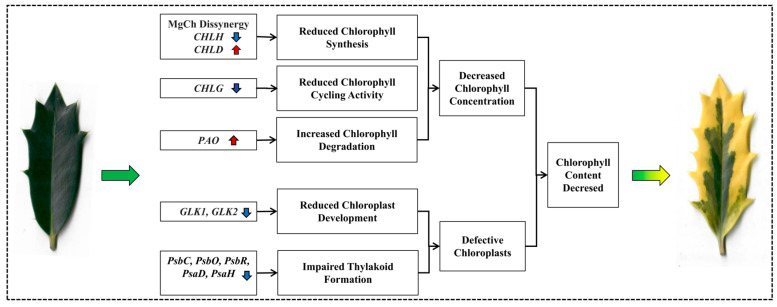
Diagram illustrating potential molecular leaf variegation in *Ilex* × ‘Solar Flare.’ A vertical red arrow denotes gene upregulation, while a vertical blue arrow indicates downregulation. The variegated leaf originates from the mutant (*I.* × ‘Solar Flare’), whereas the green leaf is from its wild-type counterpart (*I.* × ‘Conaf’).

Our comprehensive cytological, physiological, and molecular characterization provides a framework for understanding leaf variegation in *I.* × ‘Solar Flare’ (Figure 8). The evidence suggests that variegation in this cultivar results from a combination of disrupted chlorophyll biosynthesis, accelerated chlorophyll degradation, and defective chloroplast development in yellow sectors. Transcriptome sequencing identified 3510 DEGs between yellow and green sectors, with key disruptions in chlorophyll metabolism (imbalanced expression of MgCh subunits *CHLD* and *CHLH*, downregulated *CHLG*, and upregulated *PAO*) and chloroplast development (downregulation of *GLK1*, *GLK2*, and thylakoid membrane-related genes). This comprehensive characterization advances our understanding of leaf variegation in woody ornamentals and identifies promising targets for genetic manipulation to enhance this commercially valuable trait.

Future research should focus on functional validation of the identified candidate genes through transgenic approaches or virus-induced gene silencing. Additionally, comparative analyses with other variegated *Ilex* species could reveal conserved and divergent mechanisms of variegation within the genus. Exploring the genetic inheritance of variegation in ‘Solar Flare’ through crossing experiments would provide insights into the underlying genetic determinants and potential breeding applications.

## Figures and Tables

**Table 1:** Color indices of VY and VG.

Color Indices	VY	VG
L*	75.17 ± 0.30 ^a^	46.76 ± 2.20 ^b^
a*	3.37 ± 1.07 ^a^	−9.16 ± 0.61 ^b^
b*	53.99 ± 5.50 ^a^	27.02 ± 4.10 ^b^
C	54.10 ± 5.56 ^a^	28.56 ± 3.80 ^b^
H°	86.48 ± 0.76 ^b^	104.40 ± 11.70 ^a^

Note: Statistical data are shown as mean ± SD, and statistical significance (*p* < 0.05) between groups is represented by different lowercase letters. VG (the green sector of *Ilex* × ‘Solar Flare’); VY (the yellow sector of *I.* × ‘Solar Flare’).

**Table 2:** Ultrastructural examination of chloroplast ultrastructure between VY and VG.

Ultrastructural Parameters	VY	VG
Chloroplast number (per cell)	5.00 ± 0.82 ^b^	8.67 ± 1.15 ^a^
Chloroplast length (μm)	4.77 ± 1.00 ^b^	6.78 ± 0.22 ^a^
Chloroplast width (μm)	4.29 ± 0.97 ^a^	4.01 ± 0.18 ^a^
Chloroplast length/width	1.12 ± 0.08 ^b^	1.69 ± 0.03 ^a^
Starch granule number (per cell)	1.19 ± 0.19 ^a^	0.00 ± 0.00 ^b^

Note: Statistical data are shown as mean ± SD, and statistical significance (*p* < 0.05) between groups is represented by different lowercase letters. Abbreviations: VG (the green sector of *Ilex* × ‘Solar Flare’), VY (the yellow sector of *I.* × ‘Solar Flare’).

**Table 3:** Photosynthetic parameters of VY and VG.

	Parameters	VY	VG
Photosynthesis	P_n_ [μmol(CO_2_) m^−2^ s^−1^]	−2.43 ± 0.25 ^b^	2.87 ± 0.15 ^a^
	E [mol(H_2_O) m^−2^ s^−1^]	0.63 ± 0.32 ^b^	1.13 ± 0.35 ^a^
	G_s_ [mol(H_2_O) m^−2^ s^−1^]	28.33 ± 15.50 ^a^	47.33 ± 16.62 ^a^
	C_i_ [μmol(CO_2_) mol^−1^]	574.67 ± 72.14 ^a^	303.00 ± 43.31 ^b^
Chlorophyll fluorescence	Fv/Fm	0.16 ± 0.07 ^b^	0.70 ± 0.03 ^a^
	Y (II)	0.12 ± 0.08 ^b^	0.47 ± 0.02 ^a^
	NPQ	0.05 ± 0.01 ^b^	0.62 ± 0.08 ^a^
	Y (NO)	0.84 ± 0.08 ^a^	0.33 ± 0.02 ^b^
	Y (NPQ)	0.04 ± 0.01 ^b^	0.20 ± 0.02 ^a^

Note: Statistical data are shown as mean ± SD, and statistical significance (*p* < 0.05) between groups is represented by different lowercase letters. Abbreviations: C_i_ (intercellular CO_2_ concentration), E (transpiration rate), Fv/Fm (maximal quantum yield of PSII photochemistry), G_s_ (stomatal conductance), NPQ (nonphotochemical quenching), P_n_ (net photosynthetic rate), Y (II) (effective quantum yield of PSII), Y (NO) (nonregulatory energy dissipation quantum yield), Y (NPQ) (regulatory energy dissipation quantum yield), VG (the green sector of *Ilex* × ‘Solar Flare’), VY (the yellow sector of *I.* × ‘Solar Flare’).

## Data Availability

The complete dataset of Illumina sequencing results can be accessed via the NCBI Sequence Read Archive with Bioproject identifier PRJNA1035273. All data are available upon reasonable request to the corresponding author.

## Supplementary Materials

The following supporting information can be downloaded at: https://www.mdpi.com/article/10.3390/ijms26093999/s1.
