# Supplementary material for: Transcriptomic and Structural Insights into Leaf Variegation Development in Ilex × ‘Solar Flare’
Source: Int J Mol Sci. 2025 Apr 23;26(9):3999. doi: 10.3390/ijms26093999 (PMC12071917; doi:10.3390/ijms26093999)
Supplement: Supplementary file 1 [file ijms-26-03999-s001.zip › Tables S1, S2, S8 and Figure S1-S3.pdf]

Supplementary Materials

Transcriptomic and Structural Insights into Leaf Variegation Development in *Ilex* × 'Solar Flare'

Yiping Zou [1,2,3], Tao Zhuo [1], Yan Duan [4], Hong Chen [2,*], Peng Zhou [5], Mingzhuo Hao [1], Yunlong Yin [1,2] and Donglin Zhang [3,*]

1 College of Forestry, Nanjing Forestry University, Nanjing 210037, China; yiping200889@126.com (Y.Z.)
2 Institute of Botany, Jiangsu Province and Chinese Academy of Sciences (Nanjing Botanical Garden Memorial Sun Yat-Sen), Nanjing 210014, China
3 Department of Horticulture, University of Georgia, Athens, GA 30602, USA
4 College of Environment and Design, University of Georgia, Athens, GA 30602, USA; yd40901@uga.edu
5 Jiangsu Academy of Forestry, Nanjing 211153, China; zpjsslky@163.com
* Correspondence: chenhong@cnbg.net (H.C.); donglin@uga.edu (D.Z.)

Supplementary Figures

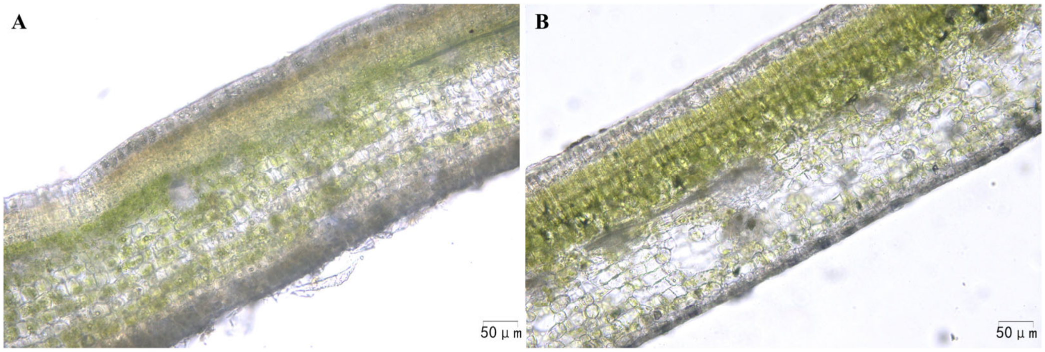

Figure S1. Cross-sectional comparison of *Ilex* × 'Solar Flare' variegated leaves (**A**) and wild-type *I.* × 'Conaf' green leaves (**B**). Scale bars = 50 μm.

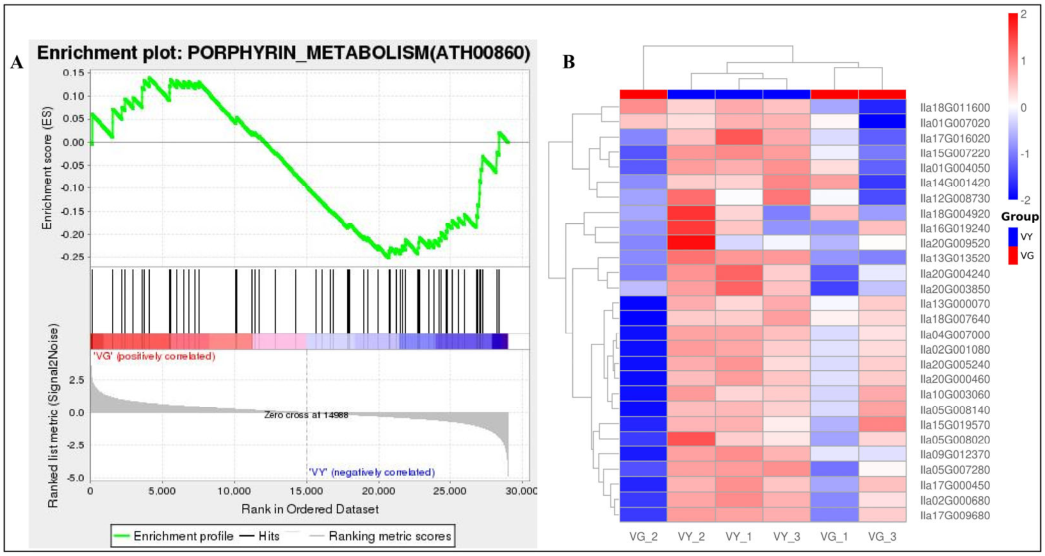

Figure S2. GSEA of the Chl biosynthesis pathway in VY. (**A**) GSEA enrichment plot in porphyrin metabolism pathway. (**B**) Hierarchical cluster analysis of GSEA enriched genes in porphyrin metabolism pathway. Red indicates up-regulated genes, and green indicates down-regulated genes. VG (the green sector of *I.* × 'Solar Flare'); VY (the yellow sector of *Ilex* × 'Solar Flare').

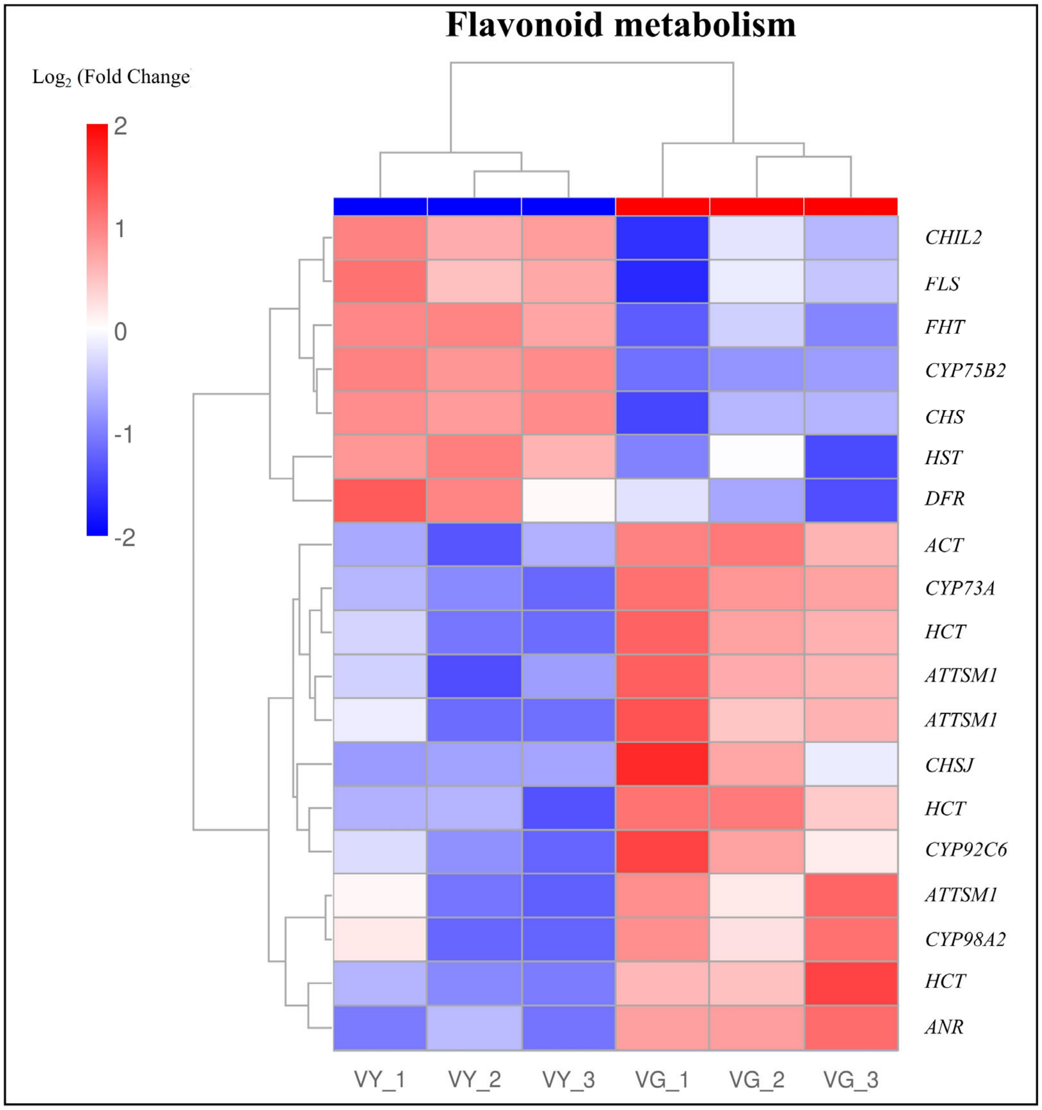

Figure S3. Hierarchical clustering and heatmap visualization of DEGs in the flavonoid biosynthesis pathway. The mean $\log_2$ (Fold Change) values for the DEGs were calculated from three biological replicates for VY and VG. Red indicates up-regulation, and blue indicates down-regulation. VG (the green sector of *I.* × 'Solar Flare'); VY (the yellow sector of *Ilex* × 'Solar Flare').

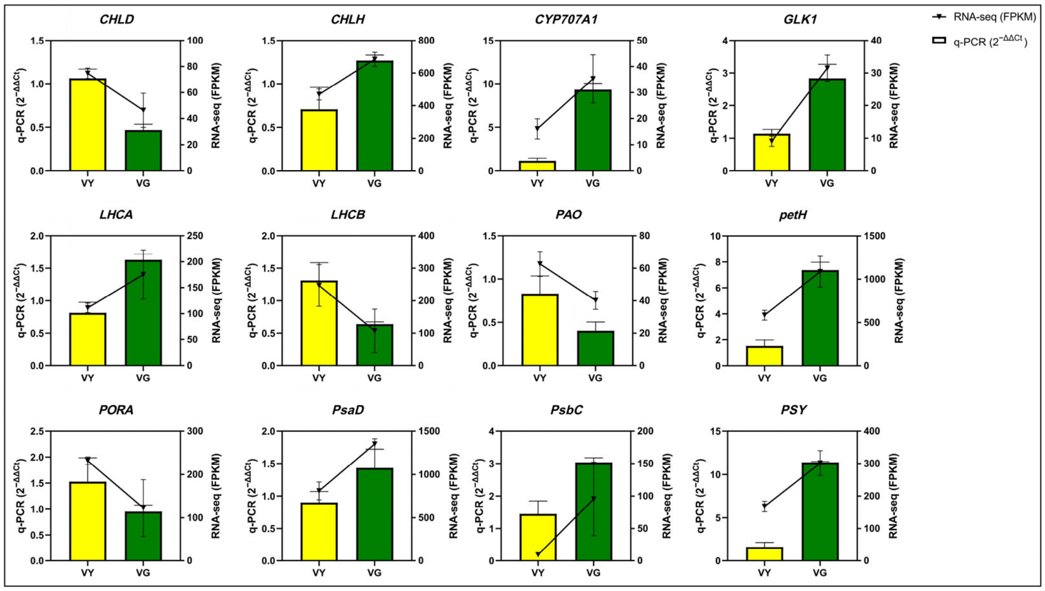

Figure S4. Validation of RNA-sequencing results through qRT-PCR analysis of 12 selected genes. VG (the green sector of *I.* × 'Solar Flare'); VY (the yellow sector of *Ilex* × 'Solar Flare').

Supplementary Tables

Table S1. Comparison of epidermal characteristics between VY and VG.

Histological Parameters	VY	VG
Stomata length (um)	25.39 ± 2.64 a	28.03 ± 5.40 a
Stomata width (um)	20.42 ± 1.78 a	26.06 ± 6.33 a
Stomata density (per um^2)	207.41 ± 36.66 b	283.59 ± 29.32 a
Closed stomata percentage (%)	84.47 ± 10.75 a	41.65 ± 3.13 b

Note: Statistical data are shown as mean ± SD, and statistical significance ($p < 0.05$) between groups is represented by different lowercase letters.

Table S2. Summary of RNA-sequencing data.

Samples	Total Reads	Clean Reads	Q20	Q30	GC Content	Mapping Rate
VG-1	42,179,038	40,898,672	96.85%	91.57%	44.16%	85.06%
VG-2	44,507,414	43,570,818	97.00%	91.80%	44.54%	86.25%
VG-3	43,401,220	42,381,148	97.10%	92.11%	44.14%	85.62%
VY-1	43,629,616	42,023,070	96.72%	91.26%	43.99%	85.25%
VY-2	44,814,204	43,369,038	97.03%	91.86%	44.00%	85.58%
VY-3	46,072,198	44,514,872	96.81%	91.40%	43.66%	84.72%
Total	264,603,690	256,757,618	96.92%	91.67%	44.08%	85.41%

Table S8. Sequences of forward and reverse primers used for qRT-PCR validation.

Gene ID	Forward Primer	Reverse Primer
GLK1(*Ila20G011400*)	ATGAGCCTTTGGGTAGCACC	GTCTGCTGGTGGTGGATGAA
PORA(*Ila17G009680*)	CCCATTGTTCAGGGCTCTGT	CACCTTCCTGGCCTTCTCTG
CHLD(*Ila09G012370*)	TGTAAAAGTCGTCCCCAAGC	GCCCCAAGTAAAAGAGCAGT
PAO(*Ila17G016020*)	AGAGCATGTGCCACAAGGTT	GTCCAGTGCTCTTGCCATCT
PSY(*Ila06G009790*)	TGTTGATGGCCCTAACGCAT	GAACGTCCTTGCCCTCTTGA
CYP707A1(*Ila10G003260*)	TTCAATGGGGTGGCCTCTTG	GCCTGAACCAGCTTCCTCAT
PsaD(*Ila11G008410*)	CCACCACGCCCTATTCG	CCAGTGCTGCCACCAAAT
LHCA(*Ila05G002490*)	GCATTCTAAACACCCCTTCA	ACCCGACATCCGTTCCTG
PsbC(*Ila0G015340*)	GGCCGGAGCAATGAACCTAT	GTCTCAGGTCCCAGAAGTGC
LHCB(*Ila15G017050*)	CTTGGGGCTCTTGGATGTGT	CCAATGGGTCGAAGCTTCCT
petH(*Ila06G008090*)	CCATCCTCCAAATCTTCTTCTC	GTTGTGACCTGGGCTCTGA
CHLH(*Ila19G011500*)	GAACATTCACGCGCTGGATC	TTGGAAGAGTAGGAGCCGGA
Actin	CCACCTACAACTCCATCAT	TTCCTTGCTCATACGATCA
